# Composite Structure Based on Gold-Nanoparticle Layer and HMM for Surface-Enhanced Raman Spectroscopy Analysis

**DOI:** 10.3390/nano11030587

**Published:** 2021-02-26

**Authors:** Zirui Wang, Yanyan Huo, Tingyin Ning, Runcheng Liu, Zhipeng Zha, Muhammad Shafi, Can Li, Shuanglu Li, Kunyu Xing, Ran Zhang, Shicai Xu, Zhen Li, Shouzhen Jiang

**Affiliations:** 1 Collaborative Innovation Center of Light Manipulations and Applications in Universities of Shandong School of Physics and Electronics, Shandong Normal University, Jinan 250014, China; 201909020549@stu.sdnu.edu.cn (Z.W.); yanyanhuo2014@sdnu.edu.cn (Y.H.); ningtingyin@sdnu.edu.cn (T.N.); 2019020507@stu.sdnu.edu.cn (R.L.); 2019020512@stu.sdnu.edu.cn (Z.Z.); 2019201014@stu.sdnu.edu.cn (M.S.); 2018020569@stu.sdnu.edu.cn (C.L.); 201809020248@stu.sdnu.edu.cn (S.L.); 201909020217@stu.sdnu.edu.cn (K.X.); 201909020114@stu.sdnu.edu.cn (R.Z.); 2 Shandong Key Laboratory of Medical Physics and Image Processing & Shandong Provincial Engineering and Technical Center of Light Manipulations, Jinan 250014, China; 3 Shandong Key Laboratory of Biophysics, College of Physics and Electronic Information, Institute of Biophysics, Dezhou University, Dezhou 253023, China; shicaixu@dzu.edu.cn

**Keywords:** hyperbolic metamaterials (HMMs), gold-nanoparticle (Au-NP) layer, SERS

## Abstract

Hyperbolic metamaterials (HMMs), supporting surface plasmon polaritons (SPPs), and highly confined bulk plasmon polaritons (BPPs) possess promising potential for application as surface-enhanced Raman scattering (SERS) substrates. In the present study, a composite SERS substrate based on a multilayer HMM and gold-nanoparticle (Au-NP) layer was fabricated. A strong electromagnetic field was generated at the nanogaps of the Au NPs under the coupling between localized surface plasmon resonance (LSPR) and a BPP. Additionally, a simulation of the composite structure was assessed using COMSOL; the results complied with those achieved through experiments: the SERS performance was enhanced, while the enhancing rate was downregulated, with the extension of the HMM periods. Furthermore, this structure exhibited high detection performance. During the experiments, rhodamine 6G (R6G) and malachite green (MG) acted as the probe molecules, and the limits of detection of the SERS substrate reached 10^−10^ and 10^−8^ M for R6G and MG, respectively. Moreover, the composite structure demonstrated prominent reproducibility and stability. The mentioned promising results reveal that the composite structure could have extensive applications, such as in biosensors and food safety inspection.

## 1. Introduction

Surface-enhanced Raman spectroscopy (SERS), as a molecular detection technology exhibiting high sensitivity, has been broadly employed for environmental monitoring, chemical monitoring, and biosensors [1,2,3,4,5]. 

The electromagnetic mechanism (EM) is of critical significance for SERS enhancement mechanisms. It is implemented using the surface plasmon of the metals to improve the electromagnetic field, elevating the SERS signal to 10^8^ or more [6,7,8,9,10,11,12]. The localized surface plasmon resonance (LSPR) is considered a typical EM. The excitation of the LSPR of metallic surfaces (such as a noble metal nanoparticle layer) results in electromagnetic field enhancement. This field enhancement has a significant effect on the probe molecules adsorbed on the metal surface, enhancing their Raman signal effectively [13]. On this basis, the researchers fabricated several different substrates [4,14].

A surface plasmon polariton (SPP) indicates a surface electromagnetic wave propagating along the planar interface between a metal and a dielectric medium. Since its electromagnetic field is confined close to the metal/dielectric interface, the electromagnetic field is improved at the interface [15]. Yinzhou Chu and Fei Zhou proposed a substrate based on nanoparticles and a metal/dielectric bilayer, demonstrating that the coupling between LSPR and a SPP can enhance the Raman signal [16,17]. Nevertheless, the enhancement for the signal is relatively weak because only a metal/dielectric bilayer supports the SPP. Adjacent SPPs are effectively coupled when the metal/dielectric bilayer is replaced with a multilayer metal/dielectric bilayer structure, leading to the generation of a bulk plasmon polariton (BPP) within the multilayer structure. A BPP is a wave capable of propagating in the bulk of the material and maintaining the properties of a propagating wave [18]. A BPP is generated by the interaction of SPPs, enabling it to be more effectively coupled with LSPR than an SPP. Afterwards, Zhen Li et al. developed a substrate with an Al nanoparticle−film system, contributing to the generation of BPPs in the ultraviolet region [19]. However, the material properties are subject to several limitations, and the gaps between the dispersed metal particles in this structure are extremely large. Consequently, electromagnetic fields are not exposed between the metal particles, reducing the effectiveness of the coupling between the LSPR and the BPP. Thus, a novel Raman substrate should be proposed to more effectively exploit the coupling between LSPR and a BPP. 

In the present study, a composite structure combining a gold-nanoparticle layer with a hyperbolic metamaterial (HMM) was formed. An HMM refers to an artificial optical metamaterial composed of a certain thickness of metal films and medium films periodically stacked [20,21,22]. It supports SPPs and BPPs and also exhibits unique dispersion characteristics [18,23,24]. In such a composite structure, an HMM consisting of a multistack of Au/Al_2_O_3_ bilayers was deposited on a PET (polyethylene terephthalate) film, and a Au-NP layer was formed and then deposited on the topmost part of the HMM. Given the momentum mismatch between an SPP and incident light [25,26], the Au-NP layer acted as a 2D grating, which could help to match the momentum conditions and successfully excite the SPP mode and the BPP mode at optical frequencies [20,27,28]. With the increase in the number of Au/Al_2_O_3_ bilayers, the BPP could facilitate the LSPR excitation of the Au-NP layer [29]; a great interaction (coupling) was identified between the BPP and LSPR, and the coupling would generate a strong local electromagnetic field (hot spots) at the nanogaps of the Au NPs, so as to greatly enhance the Raman signal for analysis. In the experiments, rhodamine 6G (R6G) acted as the probe molecule. The intensity of the Raman signal was suggested to increase, whereas the rate of increase was downregulated as the number of Au/Al_2_O_3_ bilayers increased. Next, different concentrations of R6G and malachite green (MG) were used to examine the performance of the composite structure, with the R6G and the MG concentrations ranging from 10^−6^ to 10^−10^ M and 10^−4^ to 10^−8^ M, respectively, as obtained in six periods of the composite structure under 532 nm incident light. According to the results of COMSOL simulation and SERS experiments, the composite structure exhibited excellent sensitivity and practicability.

## 2. Materials and Methods

### 2.1. Fabrication of the Au Nanoparticles 

**Figure 1:**
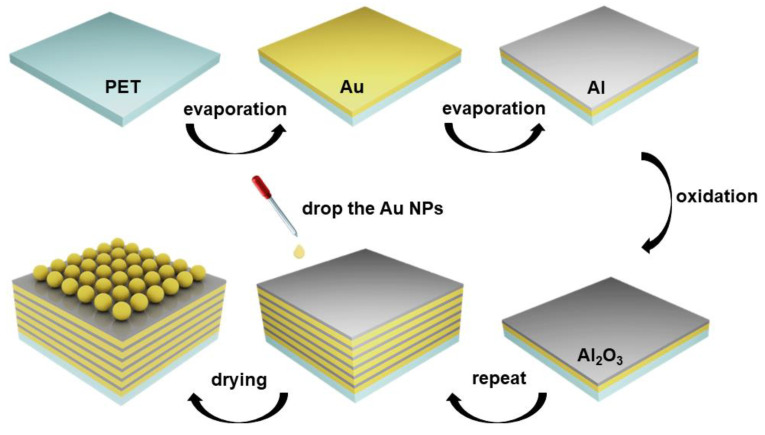
The fabrication of the composite structure substrate.

The fabrication of the composite structure substrate was illustrated in Figure 1. The Au nanoparticles were prepared with the method described in [30]. A 0.2 wt% (5 mL) chloroauric acid solution and 75 mL of deionized water were mixed and then added into a flask. The mixture was heated at 60 °C for 5 min under magnetic stirring. Subsequently, a reducing mixture of 0.0010 g of tannic acid, 0.040 g of trisodium citrate and 0.0007 g of potassium carbonate dispersed in deionized water (20 mL) was added into the flask. After the reaction was allowed to occur for one hour, the obtained solution was centrifugated at 1000 rpm for 15 min. Afterwards, the centrifugated deposit was dispersed in deionized water again for use.

### 2.2. Fabrication for the Composite Structure Substrate

First, a PET substrate with the dimensions of 10 × 10 cm^2^ was ultrasonically washed with acetone, alcohol and deionized water in a consecutive manner to annihilate the surface impurities. Subsequently, vacuum thermal evaporation was employed to deposit an 8 nm Au film and a 3 nm Al film on the PET film at the rates of 0.1 and 0.5 Å/s, respectively, under a constant pressure of 7 × 10^−5^ Pa. Additionally, the samples were oxidized in pure oxygen for 10 min to completely oxidize the Al film and form a high-quality oxide layer. The mentioned steps were repeated, and the multilayer structure in the different periods could be successfully fabricated. Next, the prepared gold nanoparticles, with hexane and ethyl alcohol at a volumetric ratio of 2:1:1, were dropped onto the substrate and then air-dried. Finally, near-uniformly distributed gold nanoparticles were fabricated successfully.

### 2.3. Characterization and SERS Experiments

A scanning electron microscope (SEM, Zeiss Gemini Ultra-55, Oberkochen, Germany) and atomic force microscopy (AFM, Park XE100, France) were employed to test the morphology of the multilayer structure and the surface topographies of the gold nanoparticles. The Raman spectroscopy was performed using a Raman spectrometer (Horiba HR Evolution, Kyoto, Japan). Furthermore, the Raman spectra were collected under the conditions of a 532 nm laser; the laser power was 0.48 mW, the gratings had 600 grooves per nanometer, a ×50 objective lens was used, and a 1 μm laser spot was used; the acquisition time was 4 s. 

## 3. Results and Discussion

**Figure 2:**
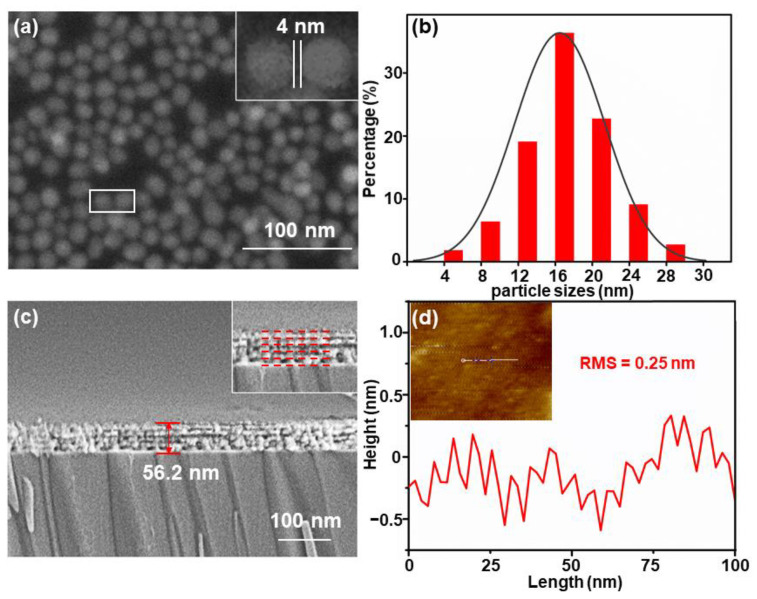
(**a**). SEM image of the Au-nanoparticle (NP) layer after drying naturally. (**b**) The distri-bution of the diameters of the Au NPs. (**c**) Cross-sectional SEM image of five-Au/Al_2_O_3_-bilayer structure. The inset shows a local-magnification image. (**d**) The root-mean-square (RMS) roughness of the Al film can be seen from the white line drawn in the inset.

After the gold nanoparticles were dropped and dried naturally, an SEM image of the Au NPs with a near-uniform distribution was taken and is illustrated in Figure 2a. It reveals that a particle layer was deposited on the top of the substrate, and the size of the gaps was nearly 4 nm. The particle size statistical distribution is presented in Figure 2b, where the Au NPs exhibited a diameter of about 16 nm, conforming to a Gaussian distribution. For instance, a cross-sectional image of five Au/Al_2_O_3_ bilayers is exhibited in Figure 2c. The actual thickness reached 56.2 nm, close to the expected thickness of 55 nm. Besides, an 8 nm Au film (light area), a 3 nm Al_2_O_3_ film (dark area) and the boundary could be observed. In the mentioned structure, the topmost Al_2_O_3_ film in the composite structure exhibited a root-mean-square (RMS) roughness of 0.25 nm (Figure 2d). As revealed from the mentioned result, the topmost Al_2_O_3_ film was smooth and could act as an effective platform for making Au NPs distribute uniformly on the top of the substrate. Meanwhile, the obtained smooth surface prevented the losses of the SPP from being scattered and exerted a more significant effect in supporting plasmonic resonance coupling [19,31]. Moreover, it was simulated with COMSOL to verify that the composite structure exhibited a great coupling.

**Figure 3:**
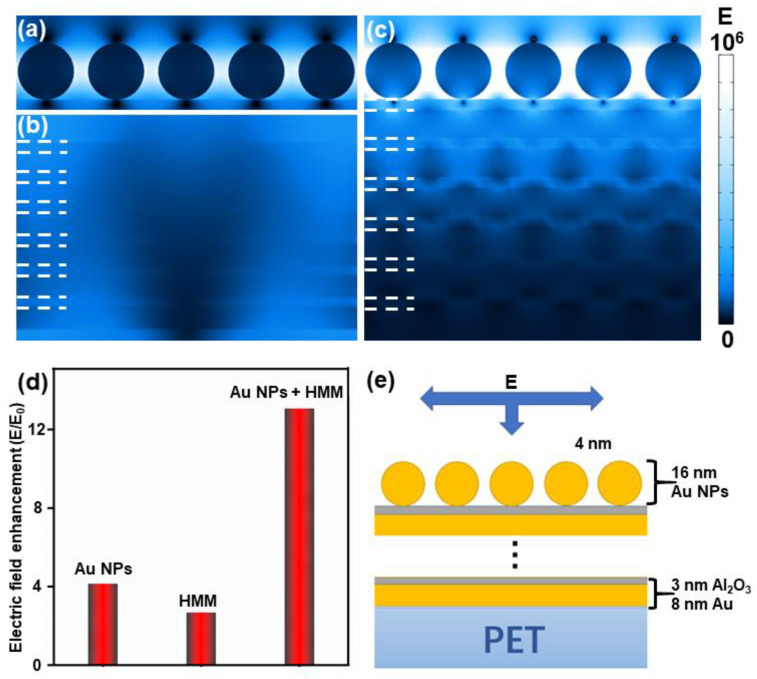
The simulation of *y–z* views of electric field distributions for (**a**) single Au-NP layer, (**b**) the hyperbolic metamaterial (HMM) consisting of six bilayers with 8 nm Au and 3 nm Al_2_O_3_, and (**c**) the combination of the Au-NP layer and HMM. (**d**) A histogram drawing a comparison of the electric field enhancement (*E*/*E*_0_) among the Au NPs, HMM and Au NPs + HMM composite structure. (**e**) The simulation setup of the composite structure substrate.

In this study, the electric field distributions of different structures were simulated using the COMSOL simulation software. All the simulations were under 532 nm incident wave polarities along the y-direction. The electric field distribution of the Au-NP layer is illustrated in Figure 3a. The diameter of the Au NP was 16 nm, and the gap reached 4 nm. Figure 3b presents the HMM structure in six periods with an 8 nm Au film and 3 nm Al_2_O_3_ film. According to the two images, both the Au-NP layer and HMM exhibited extremely weak hot-spot intensity. As indicated by Figure 3c, when the Au-NP layer was combined with the HMM, the hot spots at the gaps of the Au-NP layer exhibited strong intensity, while a great coupling was achieved inside the HMM. The results of the comparison are provided in Figure 3d. Additionally, the substrate model is presented in Figure 3e. In this study, a 3 nm Al_2_O_3_ film as the interval layer was selected since it is convenient to fabricate. Additionally, the Al_2_O_3_ film was applied as the interval layer as it can separate adjacent Au films and prevent the interaction of Au films [32]. An SPP was generated on the Au film surface when the HMM was excited. The SPPs of the adjacent films should also be well coupled to generate a strong BPP.

The skin depth of the SPP was limited when it was propagated in the metal film, and the limitation was about 30 nm for Au [33,34]. Thus, the 8 nm Au film could generate an SPP and be penetrated by the SPP, resulting in SPP coupling with at least two adjacent films. According to Figure 3c, the coupling inside the HMM could be clearly noticed, and each film was excited to varying degrees. Furthermore, the enhancement of the Au-NP layer was suggested to be relatively higher than that of the HMM since there was a weak LSPR at the gap of the Au NPs, as demonstrated by comparing the electromagnetic field intensities of the different structures. Moreover, the enhancement of the composite structure (Au NPs + HMM) was significantly higher than that of the Au-NP layer and HMM. This result can be explained, as the Au-NP layer was employed to be a 2D grating and matched the momentum conditions of the SPP; the excited SPPs propagated inside the HMM and coupled with each other, and the synergistic effect of the BPP would have significantly facilitated the LSPR excitation and the generation of strong electromagnetic fields at the gap of the Au NPs.

**Figure 4:**
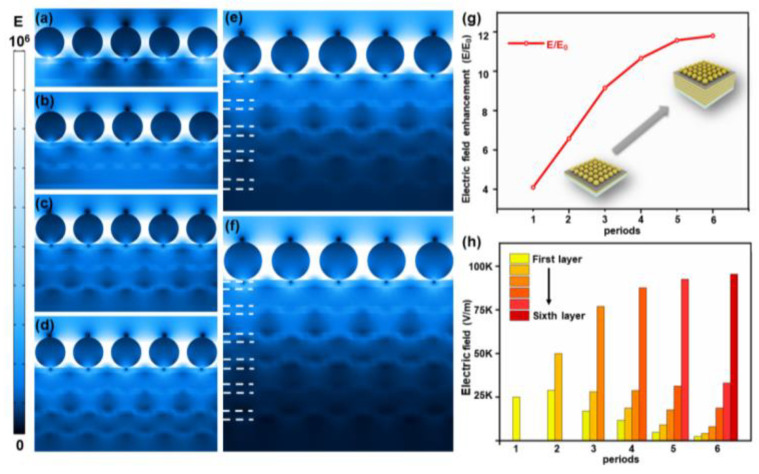
(**a**–**f**) The simulations of the electric field distribution from one to six Au/Al_2_O_3_ bilayers. (**g**) The electric field enhancement (*E*/*E*_0_) in different periods. (**h**) The electric field intensity of each Au/Al_2_O_3_ bilayer in different periods.

Figure 4a–f present the simulations of the electric field distributions of the composite structures with 1–6-bilayer Au/Al_2_O_3_ films, respectively. It can be observed that the prominent electric fields were all generated at the gaps of the gold nanoparticles. Meanwhile, the intensity of the hot spots turned stronger with increasing periods of the HMM. As revealed from the mentioned results, a synergistic effect was exerted for hot spots along the vertical direction, inducing a strong coupling at the nanogap region. Such an effect was closely related to the coupling between the LSPR and BPP, which would have enhanced the localized electromagnetic field intensity on the top of the multilayer structure. Notably, Figure 4g was generated by collecting the information from the simulation images, indicating that the intensity of the hot spots increased while the rate of increase was lowered as the number of Au/Al_2_O_3_ bilayers was elevated. This phenomenon can be explained as follows: the LSPR of the Au NPs would have absorbed the energy partially when the substrate was under the incident light condition [17], and part of the energy was absorbed by the multilayer structure HMM. When the SPP mode was excited, part of the incident light energy was converted to an evanescent field appearing on each surface of the Au film, and the energy was partially transferred to the SPP [35], making the SPP propagated on the Au surface. Since the SPP was decaying in the propagation direction, there was an energy loss in this process [25,34]. Accordingly, the deeper the incident light penetrated, the stronger the energy losses. Additionally, the limited energy of the incident light excited a limited number of Au/Al_2_O_3_ bilayers to support the SPP because each Au/Al_2_O_3_-bilayer-supporting SPP mode would have absorbed energy, causing the intensity of the BPP to be impacted. Moreover, the excited SPP achieved little energy when the incident light was penetrated to the bottom film, hindering it from being coupled well with adjacent SPPs. Furthermore, it was difficult for the bottom SPP to interact directly with the top SPP. Thus, the bottom excited SPP would have exerted less influence on the top SPP and the enhancing effect with the increase in the number of the Au/Al_2_O_3_ bilayers.

Figure 4h presents the electric field intensity of each Au/Al_2_O_3_ bilayer in different periods. It can be observed that the electric field intensity of the topmost Au/Al_2_O_3_ bilayer interlayer was always the strongest, and the other bilayers’ intensities were diminishing with increasing depth. The mentioned result can be explained by the coupling between the SPPs. Specifically, the number of SPPs participating in coupling increased as the number of Au/Al_2_O_3_ bilayers increased. Such interaction in the vertical direction led to the strongest SPP in the topmost bilayer. Moreover, the intensity of the topmost SPP tended to be enhanced, suggesting that it absorbed more energy, resulting in less and less energy transferred to the lower layers. Thus, for the SPP, the closer to the bottom, the less energy will be available. Furthermore, the increase rate for the SPP intensity on the topmost part was lowered as the periods increased because the absorption of energy causing the weak SPP at the bottom could not significantly improve the topmost SPP. 

**Figure 5:**
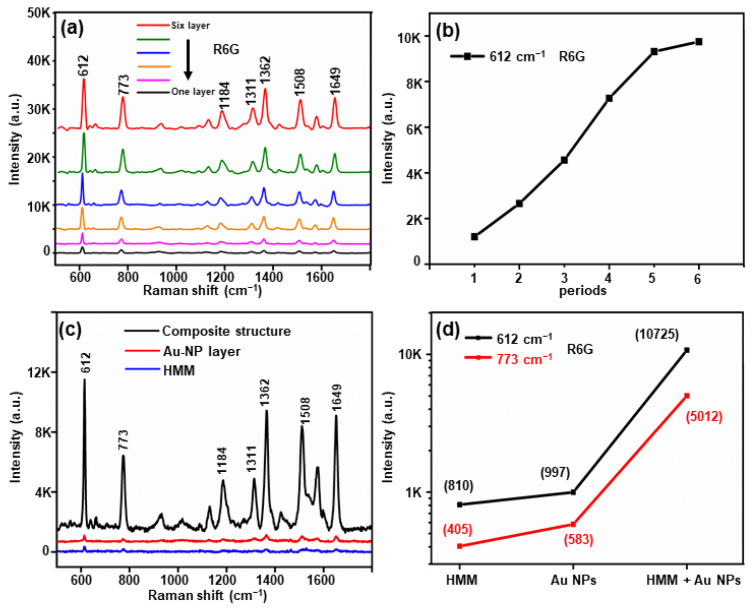
(**a**) The Raman signal of 10^−6^ M rhodamine 6G (R6G) on HMM, Au-NP layer, and composite structure with six Au/Al_2_O_3_ bilayers. (**b**) The Raman signal intensity of characteristic peak at 612 cm^−1^ in different periods. (**c**) The relationship diagram of the peak intensity of the R6G Raman spectra at 612 and 773 cm^−1^. (**d**) The Raman spectra of 10^−6^ M R6G from the different structures.

R6G as a probe molecule was chosen to measure the SERS performance of the composite structure substrate more specifically. In the experiment shown in Figure 5a, 10^−6^ M R6G was dropped on the top of the substrate and air-dried to evaluate the SERS performance of the composite structure in different periods. Figure 5b shows the Raman signal intensity of the characteristic peak 612 cm^−1^ in the different periods. The peak at 612 cm^−1^ is a Raman-characteristic peak with high enhancement, which can reflect a good interaction between the probe molecule and the substrate [36]. It can be observed that the increase rate for the Raman signal slowed down as the number of Au/Al_2_O_3_ bilayers increased. The mentioned results are consistent with the theoretical simulation, and the difference can be attributed to the fact that the simulated figure (Figure 4g) is an idealized model, while our substrate had a small number of nanoparticles with a nonuniform distribution. Figure 5c presents the Raman spectra of the 10^−6^ M R6G on the HMM, single Au-NP layer, and composite structure with six Au/Al_2_O_3_ bilayers. The characteristic Raman peaks of R6G at 612, 773, 1184, 1311, 1362, 1508 and 1649 cm^−1^ were clearly observed for the composite structure, and the signal from the composite structure exhibited a noticeably higher intensity compared to those for the Au-NP layer and HMM. The signal intensity of the Raman peaks at 612 and 773 cm^−1^ of R6G in an identical scenario is illustrated in Figure 5d to compare the SERS properties of the different structures. As revealed from the calculation of the intensity of the characteristic peak, the intensity of the peak at 612 cm^−1^ measured for the composite structure was approximately 10.8 times that measured for the Au-NP layer and nearly 13.2 times that measured for the HMM. Furthermore, the peak intensity at 773 cm^−1^ measured for the composite structure was about 8.6 times that measured for the Au-NP layer and nearly 12.4 times that measured from the HMM, demonstrating that the coupling between the Au-NP layer and HMM exerted a significant enhancing effect. 

**Figure 6:**
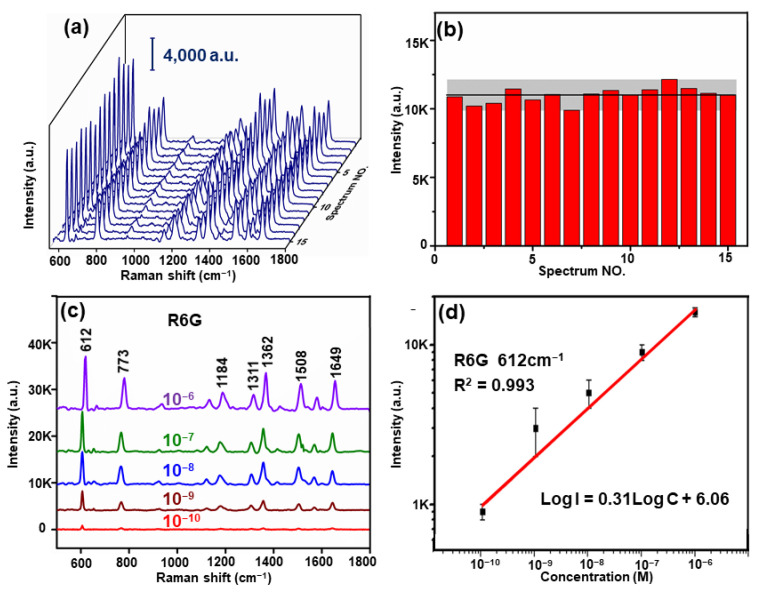
(**a**). The Raman signal of the 10^−6^ M R6G collected randomly from 15 spots in 15 composite substrates with six Au/Al_2_O_3_ bilayers. (**b**) The intensity of 612 cm^−1^ peak for the composite structures. (**c**) The SERS spectra of R6G at concentrations ranging from 10^−6^ to 10^−10^ M. (**d**) The calibration curve of the normalized Raman intensity at 612 cm^−1^ versus R6G of different concentrations.

Additionally, reproducibility was considered to be an essential indicator for examining the SERS substrate performance. As shown in Figure 6a, 15 spots from composite structures were taken randomly, and the Raman signal was collected for 10^−6^ M R6G. No significant fluctuations for each characteristic peak of R6G were observed from the data. The distribution histogram of the intensity of the characteristic peak at 612 cm^−1^ for R6G is provided in Figure 6b, where the black horizontal line represents the average intensity of the 612 cm^−1^ peak for SERS from the mentioned 15 positions, and the gray area indicates the fluctuation of the SERS signal. The relative standard deviation (RSD) of the peak intensity for R6G at 612 cm^−1^ was 4.7%, confirming that the composite structure had excellent reproducibility.

Next, R6G, as the probe molecule, was employed to measure the detection performance of the composite structure. When the probe molecules were dropped on the composite structure, they would have been gathered at the nanogaps of the Au NPs spontaneously, and the Raman signal was enhanced effectively. The SERS spectra of R6G at concentrations ranging from 10^−6^ to 10^−10^ M are illustrated in Figure 6c, where the characteristic peaks could still be clearly observed at 10^−10^ M. In the mentioned concentration range, quantitative detection could be conducted, and the variation of the intensity could be quantitatively expressed using an empirical equation for the concentration (Log *I* = 0.31Log *C* + 6.06, for R6G), where *I* denotes the intensity of the SERS signal, *C* represents the concentration of R6G, and the coefficients of determination (*R*^2^) are 0.993. The LOD of R6G was calculated as LOD = 3.3(SD/*S*), where SD indicates the standard deviation of the response, and *S* is the slope of the calibration curve; the result was nearly 7.9 × 10^−11^ M, satisfying practical detection use. Furthermore, the electromagnetic enhancement factors (*EFs*) were calculated by [37,38]

(1) $$EF=\frac{I_{SERS}/N_{SERS}}{I_{RS}/N_{RS}}$$

where *I_SERS_* and *I_RS_* represent the peak intensities of the SERS spectrum and normal Raman spectrum, respectively; *N_SERS_* and *N_RS_* denote the numbers of the molecules in the laser spot regions [39]. The intensity of the peak at 612 cm^−1^ for the composite structure reached 11,725, and the intensities for 10^−6^ M R6G for the single Au-NP layer and HMM were 997 and 810, respectively. Additionally, the intensity of the identical peak on the blank PET substrate was 750, and the concentration reached 10^−2^ M. The *EF* of the composite structure substrate was calculated as 1.56 × 10^5^. Likewise, the *EFs* of the single Au-NP layer substrate and HMM substrate were determined as 1.33 × 10^4^ and 1.08 × 10^4^, respectively, which were significantly lower than the former. The mentioned result was attributed to the coupling between the Au-NP layer and HMM. Specifically, the enhancing effect was very strong when they were combined, and the effect was weak when they were separated.

**Figure 7:**
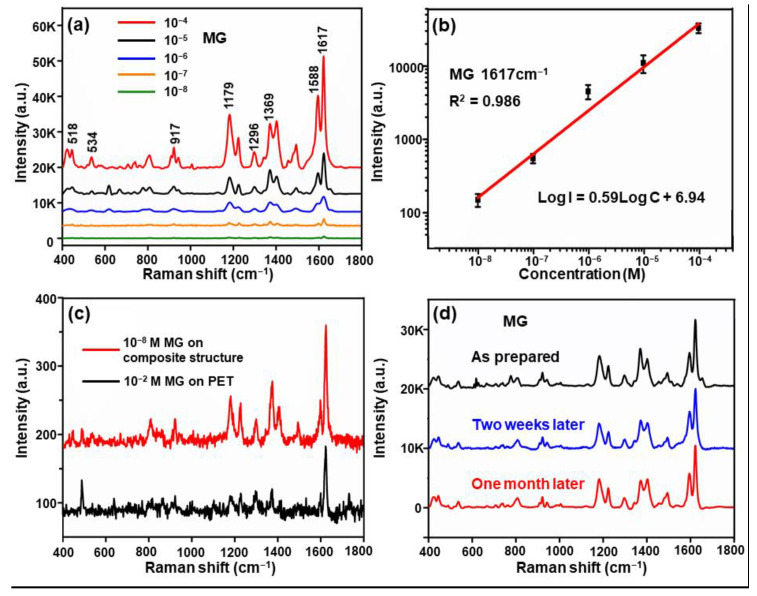
(**a**) SERS spectra acquired from 10^−4^ to 10^−8^ M malachite green (MG) deposited on the composite structure substrate displaying six Au/Al_2_O_3_ bilayers. (**b**) The calibration curve of the normalized Raman intensity at 1617 cm^−1^ versus MG of different concentrations. Error bars indicate standard deviations from 10 spectra. (**c**) The Raman spectra for 10^−8^ M MG collected for the composite structure, and 10^−2^ M obtained for PET for comparison. (**d**) The long-term stability for the MG SERS spectra collected from the composite structure substrate stored for the different periods.

The detection capability of the composite structure at a low concentration was investigated with MG as the probe molecule. The SERS spectra of MG at concentrations ranging from 10^−4^ to 10^−8^ M are presented in Figure 7a. Similarly, the 1617 cm^−1^ peak of MG could still be observed at the concentration of 10^−8^ M, demonstrating that the detection limit of the composite structure of MG was 10^−8^ M in magnitude. Figure 7b shows the standard calibration plot of the SERS signal at 1617 cm^−1^, while the peak at 1617 cm^−1^ was also the peak with higher enhancement, which is indicative of an interaction between the probe molecule and the substrate [36]. The intensity was quantitatively changed using the empirical equation for the concentration (Log *I* = 0.59Log*C* + 6.94). The coefficients of determination (*R*^2^) reached 0.986 for 1617 cm^−1^, exhibiting a great linear response in the range of concentrations. Additionally, the Raman spectra of the 10^−2^ and 10^−8^ M MG acquired from the blank PET substrate and composite structure substrate in six periods are exhibited in Figure 7c. The EF was calculated as 1.7 × 10^6^. Moreover, the long-term stability, where the intensity of the SERS signal was stable, showed an advantage of the composite structure. In this study, different positions were randomly selected from the composite structure; then, the SERS spectra were immediately collected. Subsequently, the substrate was exposed to the air, and the SERS spectra were obtained again two weeks and one month later, respectively (Figure 7d). According to this figure, the SERS signal barely decreased even after one month. This is due to the fact that the gold nanoparticles were stable in the air, and the Al_2_O_3_ film exhibited a high antioxidant property and kept the substrate stable for a long time.

## 4. Conclusions

In this study, a Au-NP/HMM composite structure was successfully formed. The Au NPs acted as a 2D grating, capable of efficiently exciting the SPP mode and the BPP mode. The LSPR excitation was facilitated by the excited BPP. Accordingly, a strong electromagnetic field was generated at the nanogaps of the Au NPs. Throughout the simulations, the results displayed a tendency consistent with the experimentally achieved results. The Raman signal acquired from the composite structure was noticeably stronger than that collected from the HMM and Au-NP layer and exhibited great sensitivity. Moreover, the intensity of the hot spots and the increase rate for the Raman signal were suggested to be lowered with an increase in the Au/Al_2_O_3_ bilayers. The mentioned factors could be attributed to energy absorption and losses. Additionally, low concentrations of R6G and MG were employed to examine the detection capacity of the composite structure, with lower-limit concentrations of 10^−10^ and 10^−8^ M, respectively. Furthermore, the reproducibility and long-term stability performance of the composite structure were determined, generating excellent results. As revealed from the mentioned promising results, the composite structure could exploit the value of SERS and contribute to the in-depth development of biosensors and food safety inspection.

## Data Availability

Data is contained within the article.
